# The Nasopharyngeal Carcinoma and Its Effect on the Infectious Eye Disease: A Nationwide Cohort Study

**DOI:** 10.3390/cancers14235745

**Published:** 2022-11-23

**Authors:** Chia-Yi Lee, Ming-Ju Hsieh, Jing-Yang Huang, Yih-Shiou Hwang, Chao-Kai Chang, Hung-Chi Chen, Shun-Fa Yang

**Affiliations:** 1 Institute of Medicine, Chung Shan Medical University, Taichung 40201, Taiwan; 2 Nobel Eye Institute, Taipei 100008, Taiwan; 3 Department of Ophthalmology, Jen-Ai Hospital Dali Branch, Taichung 41265, Taiwan; 4 Oral Cancer Research Center, Changhua Christian Hospital, Changhua 50006, Taiwan; 5 Graduate Institute of Biomedical Sciences, China Medical University, Taichung 40402, Taiwan; 6 Department of Post-Baccalaureate Medicine, College of Medicine, National Chung Hsing University, Taichung 40227, Taiwan; 7 Department of Medical Research, Chung Shan Medical University Hospital, Taichung 40201, Taiwan; 8 Department of Ophthalmology, Chang Gung Memorial Hospital, Taoyuan City 33305, Taiwan; 9 Department of Medicine, College of Medicine, Chang Gung University, Taoyuan 33302, Taiwan; 10 Department of Optometry, Yuanpei University of Medical Technology, Hsinchu 30015, Taiwan; 11 Department of Optometry, Da-Yeh University, Chunghua 51500, Taiwan; 12 Center for Tissue Engineering, Chang Gung Memorial Hospital at Linkou, Taoyuan City 33305, Taiwan

**Keywords:** nasopharyngeal carcinoma, orbital cellulitis, infectious keratitis, infection, epidemiology

## Abstract

**Simple Summary:**

The nasopharyngeal carcinoma (NPC) is a common cancer in the Asian region, and some ocular complications have thus been described. Thus, we aim to evaluate the possible relationship between NPC and following orbital cellulitis and infectious keratitis. We found that the NPC could correlate to a higher rate of following infectious keratitis and orbital cellulitis. Lower threshold for referral to ophthalmic department is suggested for NPC patients if any ocular symptom has developed.

**Abstract:**

The purpose of this study is to investigate the potential correlation between nasopharyngeal carcinoma (NPC) and both infectious keratitis and orbital cellulitis. The retrospective cohort study used the National Health Insurance Research Database (NHIRD) as a data source. A total number of 4184 patients with NPC diagnosis were selected and matched to 16,736 non-NPC patients via the propensity-score matching (PSM). The main outcomes are the development of infectious keratitis and orbital cellulitis according to diagnostic codes and related medications. The Cox proportional hazard regression was adopted to estimate the adjusted hazard ratio (aHR) and 95% confidence interval (CI) of outcomes between the two groups. A total of 35 and 19 episodes of orbital cellulitis occurred in the NPC and non-NPC groups and the aHR was significantly higher in the NPC group (aHR: 1.34, 95% CI: 1.23–1.46, *p* = 0.0024). There were 2185 and 659 events of infectious keratitis in the NPC and non-NPC groups, and the NPC group revealed a significantly higher aHR than non-NPC group (aHR: 1.34, 95% CI: 1.23–1.46, *p* < 0.0001). Besides, the cumulative probability of infectious keratitis was significantly higher in the NPC group than the non-NPC group (*p* < 0.0001). The other risk factors of infectious keratitis include age from 20 to 30 years old, allergic respiratory diseases, allergic dermatological diseases, and external eye diseases (all *p* < 0.0001). In subgroup analyses, both the incidences of infectious keratitis (aHR: 1.33, 95% CI: 1.21–1.47) and orbital cellulitis (aHR: 2.36, 95% CI: 1.27–4.39) were significantly higher than the non-NPC group. The incidence of infectious keratitis was significantly higher in NPC patients without radiotherapy (aHR: 1.40, 95% CI: 1.26–1.55) compared to non-NPC population, while the rate of orbital cellulitis was similar between the NPC patients without radiotherapy (aHR: 0.76, 95% CI: 0.27–2.14) and non-NPC population. In conclusion, the existence of NPC associates with higher incidence of infectious keratitis which increases with NPC period, and the radiotherapy may be account for the higher rate of orbital cellulitis in NPC population.

## 1. Introduction

The nasopharyngeal carcinoma (NPC) is a carcinoma with epithelial origin around the nasopharyngeal area [1]. The prevalent area of NPC is the East and Southeast Asia region [2], and the prevalence of NPC is about 3.0 per 100,000 individuals in the Chinese population which is 7-folds higher than the prevalence in the Caucasian population [3]. The tobacco consumption, Epstein–Barr virus infection and human papillomavirus infection are the established risk factor for the NPC occurrence [1,3,4]. The current managements include chemotherapy and radiotherapy, and about one-fifth of patients experience recurrence [5,6]. The median of survival interval in NPC was 3 years in the late stage and the divorce status showed a lower survival rate [7,8].

The NPC and associated management like radiotherapy could cause injury to the adjunct tissue [9]. The lymphadenopathy-induced cervical mass is a common complication of NPC [9], and the chronic rhinosinusitis was associated with the presence of NPC [10]. Besides, the development of NPC and radiotherapy for the NPC could lead to the auditory system damage such as sensorineural hearing loss and otitis media with effusion [9,11]. In addition to the above disorders, the swallowing difficulty, meningitis, cavernous sinus thrombosis, skull base osteoradionecrosis, and other central nervous system disorders have been demonstrated after the NPC development and the receipt of radiotherapy [12,13].

The ocular region locates near to the nasopharyngeal area and the NPC-related sequelae occurred in the ocular region frequently [14]. According to previous research, the orbital invasion in all the NPC individuals was approximately 1.1 percent, which accounts for nearly 50 percent of ophthalmic complications in NPC progression [15]. Additionally, the radiotherapy for the NPC management could contribute to cataract formation and radiation retinopathy with reduced visual acuity [16]. Still, there is rare evidence that whether the NPC can increase the rate of eye infection. Because the orbital invasion of NPC can cause the destruction of orbital tissue and proptosis [15], the infectious eye disease may occur frequently after the damage.

Consequently, the purpose of our study is to evaluate the potential relationship between the NPC and subsequent infectious eye disease including the orbital cellulitis and infectious keratitis via the usage of the National Health Insurance Research Database (NHIRD) of Taiwan. Several confounders including the effects of radiotherapy and chemotherapy for the two infectious eye diseases were included in our multivariable analysis.

## 2. Materials and Methods

### 2.1. Data Source

The NHIRD in Taiwan contains the claimed data of health insurance for nearly all the Taiwanese which is about 23 million persons. The NHIRD includes medical data of all individuals from 1 January 2000 to 31 December 2016. The available data of NHIRD involving International Classification of Diseases, Ninth Revision (ICD-9) diagnostic code, medical department type, sex, age, place of inhabitant, education level, image exam code, laboratory exam code, and procedure codes, which include the surgery and international ATC codes for medicines. In our study, we utilised the longitudinal health insurance database (LHID) 2000 version, a sub-database from NHIRD, for the data collection and analyses. The LHID 2000 contains approximately two million patients who were randomly selected from the NHIRD in 2000, and the data in NHIRD are also available in LHID 2000. In addition, the individuals in the LHID 2000 were followed with the same period as in NHIRD, which indicates an interval from the 1 January 2000 to the 31 December 2016.

### 2.2. Patient Selection

We defined patients as NPC with the following criteria: (1) receipt of specific ICD-9 diagnostic codes of NPC, (2) the performance of biopsy and sinoscopy before or at the same day of the NPC diagnosis, (3) the performance of Epstein–Barr virus DNA exam before t or at the same day of the NPC diagnosis, (4) follow up period for more than 3 years, and (5) the diagnosis of NPC was established by an otorhinolaryngologist. We set the index date as the date one year after the diagnosis of NPC. To enhance the homogeneity of the study population, we applied these exclusion criteria: (1) those younger than 20 years old, (2) presence of blindness before index date, (3) the arrangement of eyeball removal before the index date, (4) presence of ocular tumour before index date, (5) the arrangement of corneal transplantation before index date, (6) the occurrence of outcome before the index date, (7) the participant died before the index date. After this procedure, one NPC patients were age- and sex-matched to 8 non-NPC patients, and these participants were then matched by propensity-score match (PSM) with specific demographic data and systemic co-morbidities (listed in the following section) via a 1:4 ratio between NPC and non-NPC population. A total of 4184 NPC individuals and 16,736 non-NPC individuals were included in the NPC group and non-NPC group in the current study after all of the procedures.

### 2.3. Main Outcome Measurement

The main outcome is the presence of both the orbital cellulitis and infectious keratitis. The following criteria need to be fulfilled for the orbital cellulitis: (1) the presence of orbital cellulitis-associated ICD-9 diagnostic codes, (2) the arrangement of computed tomography before or on the day of orbital cellulitis diagnosis, (3) the application of oral and topical antibiotic after the orbital cellulitis diagnosis, and (4) the orbital cellulitis diagnosis was entered by an ophthalmologist. Additionally, the subsequent criteria is warranted for the infectious keratitis: (1) the presence of infectious keratitis-associated ICD-9 diagnostic codes, (2) the performance of silt lamp exam and corneal fluorescein stain before or on the day of infectious keratitis diagnosis, (3) the application of topical antibiotic after the infectious keratitis diagnosis, and (4) the infectious keratitis diagnosis was entered by an ophthalmologist. Of note, we only accounted the patients diagnosed with orbital cellulitis or infectious keratitis one year after the index date as outcome complement.

### 2.4. Demographic and Co-Morbidity Covariates

To diminish the effect of potential predisposing factors of orbital cellulitis and infectious keratitis, we estimated and adjusted the influence of following parameters in our multivariable analyses: age, sex, urbanization of habitat, hypertension, diabetes mellitus (DM), stable coronary arterial disease (CAD), cerebrovascular disease, hyperlipidemia, allergic respiratory diseases, rheumatic disease, allergic dermatological diseases, allergic otolaryngologic diseases, and external eye diseases. The allergic respiratory diseases referred to bronchitis, asthma, pneumoconiosis, and hypersensitivity pneumonitis. The allergic dermatological diseases referred to atopic dermatitis, contact dermatitis and urticaria. The allergic otolaryngologic diseases referred to allergic rhinitis, chronic rhinitis, chronic sinusitis and nasal polyp. Additionally, the external eye diseases referred to dry eye disease, trichiasis, blepharitis, exposure keratopathy, and thyroid eye disease. To make sure the systemic diseases affect the condition of each participant long enough, only the systemic morbidities persisted longer than two years were considered in our analysis model. Additionally, we recorded the chemotherapy, radiotherapy, and the field of radiotherapy in the NPC population. We tracked our participants until the outcome complement, quit the National Health Insurance Program or to the final day of NHIRD/LHID 2000 which implies 31 December 2016.

### 2.5. Statistical Analysis

We conducted SAS version 9.4 (SAS Institute Inc., Cary, NC, USA) for the statistical analyses. Firstly, the absolute standardized difference (ASD) was applied to illustrate the frequency of basic characteristics between the NPC and non-NPC population. Specifically, the ASD value higher than 0.1 seemed to be a significant difference. Then, we adopted Poisson regression for the incidence ratio of orbital cellulitis and infectious keratitis and applied Cox proportional hazard regression for the adjusted hazard ratios (aHR) and 95% confidence intervals (CI) of orbital cellulitis and infectious keratitis between the NPC and non-NPC groups. The Cox proportional hazard regression incorporates the effect of both the demographic data, systemic co-morbidities, and external eye diseases in the analysis, and the influence of potential confounders on the orbital cellulitis and infectious keratitis were calculated via Cox proportional hazard regression. We pictured the Kaplan–Meier curve to reveal the cumulative probability of infectious keratitis between the NPC and non-NPC groups, and we utilized the log rank test for determining the significance between the NPC and non-NPC populations. For the potential effect of chemotherapy, we divided the NPC group into patients who received chemotherapy and patients did not receive chemotherapy, then we used Cox proportional hazard regression to produce the aHR of infection eye diseases among different groups, including non-NPC population. Similarly, we categorized the NPC group into those received high-dose radiotherapy, those received low dose radiotherapy and those without radiotherapy, and we compared the difference of infection eye diseases among them plus non-NPC population according to the Cox proportional hazard regression and associated aHR. The watershed of high dose radiotherapy was set at 72 field. The statistical significance was regarded as *p* < 0.05 in the current study, and a *p* value lower than 0.0001 was described as *p* < 0.0001.

## 3. Results

The basic information between the NPC and non-NPC groups is presented in Table 1. The distribution of age, sex, and urbanization were similar between the two groups due to the PSM process. The most frequent co-morbidity was external eye diseases which developed in 851 (20.34%) NPC individuals and the most frequent systemic co-morbidity in 1112 (26.58%) and 4070 (24.32%) patients in the NPC and non-NPC groups. However, the distribution of all the co-morbidities between the two groups was similar (Table 1).

**Figure 1:**
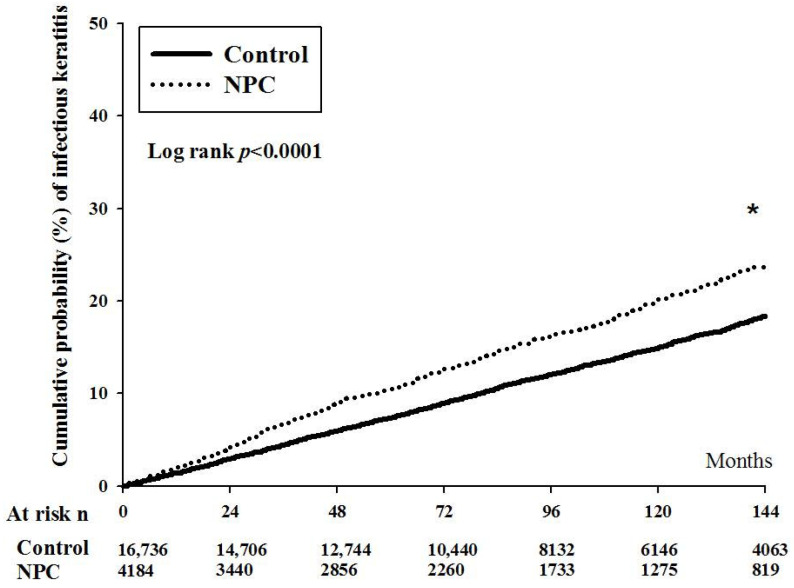
The cumulative probability of infectious keratitis between nasal pharyngeal carcinoma and non-nasal pharyngeal carcinoma groups. NPC: nasal pharyngeal carcinoma, N: number, PSM: propensity-score match. * denotes significant difference between the two groups.

After the whole interval of LHID 2000, there were 35 and 19 episodes of orbital cellulitis in the NPC and non-NPC groups. The aHR of orbital cellulitis was significantly higher in the NPC group after adjusting multiple potential confounders (aHR: 2.24, 95% CI: 1.17–3.98, *p* = 0.0041) (Table 2). On the other hand, there were 2185 and 659 events of infectious keratitis in the NPC and non-NPC groups, respectively. The NPC group also revealed a significantly higher aHR when compared to the non-NPC group (aHR: 1.29, 95% CI: 1.16–1.39, *p* < 0.0001) (Table 2). Moreover, the cumulative probability of infectious keratitis was significantly higher in the NPC population than the non-NPC population (*p* < 0.0001) (Figure 1).

For the other risk factors of infectious keratitis, the patients with age from 20 to 30 showed a significantly higher rate of infectious keratitis than the patients aged 30 to 40 (*p* = 0.0003). The other risk factors of infectious keratitis include allergic respiratory diseases, allergic dermatological diseases, and external eye diseases (all *p* < 0.0001) (Table 3). On the contrary, the male sex (*p* < 0.0001) and residence in sub-urban area (*p* = 0.0018) were protective factors for the development of infectious keratitis (Table 3). About the orbital cellulitis, no significant risk or protective factors other than the NPC were found in the multivariable analysis (all *p* > 0.05) (Table 4).

In the subgroup analyses, the NPC without chemotherapy revealed a significantly higher incidence of infectious keratitis (aHR: 1.33, 95% CI: 1.21–1.47) and orbital cellulitis (aHR: 2.36, 95% CI: 1.27–4.39) than the non-NPC group. Additionally, the NPC patients with chemotherapy showed similar incidence of infectious keratitis (aHR: 1.07, 95% CI: 0.90–1.28) and orbital cellulitis (aHR: 1.03, 95% CI: 0.37–2.87) (Table 5). About the radiotherapy-based analyses, the incidence of infectious keratitis in the NPC patients without radiotherapy was significantly higher than that in the non-NPC group (aHR: 1.40, 95% CI: 1.26–1.55), and the NPC patients with low dose radiotherapy (aHR: 0.79, 95% CI: 0.64–0.97) or high dose radiotherapy (aHR: 1.11, 95% CI: 0.90–1.36) did not own higher incidence of infectious keratitis compared to the NPC patients without radiotherapy. On the other hand, the incidence of orbital cellulitis was similar between NPC without radiotherapy (aHR: 0.76, 95% CI: 0.27–2.14) and non-NPC population, and both the NPC with low dose radiotherapy and high dose radiotherapy demonstrated a significantly higher rate of orbital cellulitis than the NPC patients without radiotherapy (both 95%CI lower limits higher than 1) (Table 6).

## 4. Discussion

In the current study, the incidence of infectious keratitis was higher incidence in the patients with NPC, whether the radiotherapy and chemotherapy were arranged or not. Moreover, the infectious keratitis demonstrated a higher cumulative probability in the NPC group than the non-NPC group. The other risk factors for infectious keratitis development included young age, allergic diseases, and external eye diseases. On the contrary, the NPC patients with radiotherapy showed a higher rate of orbital cellulitis than the NPC patients without radiotherapy and non-NPC group.

The presence of NPC would increase the inflammation response and causes several ophthalmic disorders [15]. The local inflammation disease like chronic rhinosinusitis and allergic rhinitis was observed in individuals developed NPC in following times [17]. In fact, the genetic polymorphism interleukins can influence the development and susceptibility of NPC [18]. Besides, the NPC will lead to orbital bone destruction and the swelling of adjunct soft tissue including orbital apex and extraocular muscles [15]. Additionally, the orbital involvement of NPC would also contribute to the change of orbital structure [14]. In previous research, the proptosis has been reported in 92.3 percent of patients with NPC and orbital involvement [14]. In severe cases, the proptosis may be associated with eyeball protrusion and restriction of extraocular muscle movement, which case surgery may be necessary [19,20]. Concerning the optic nerve, the mass effect of the NPC contributed to the compressive optic neuropathy and visual impairment [21]. About the infectious eye disease, the orbital cellulitis referred to both the inflammatory and infectious reaction in the orbit behind the orbital septum [22]. The major etiology of orbital cellulitis is bacterial infection like the Haemophilus influenzae type B while the fungal infection, inflammatory disorders, and malignancy had been proven to cause orbital cellulitis [23]. The infection at nearby region like the sinus and upper respiratory tract area can contribute to the development of orbital cellulitis, orbital abscess, or subperiosteal abscess [22]. On the other side, the infectious keratitis is also a disease with inflammatory and infectious manifestations [24]. Similar to the orbital cellulitis, the microorganism plays a dominant character in the occurrence of infectious keratitis [25], and the most common microorganisms in infectious keratitis are coagulase negative staphylococci, Streptococcus pneumoniae, and Staphylococcus aureus [26]. Besides, approximately 16 percent of patients with infectious keratitis exhibited corneal surface exposure [27]. Because the NPC can destruct orbital tissue and contribute to subsequent inflammation and eyeball protrusion, we think it may be possible that the existence of NPC would increase the probability of orbital cellulitis and infectious keratitis. The results of the current study support our concept.

The presence of NPC in the current study is associated with the development of following infectious keratitis. To our knowledge, few studies reported such correlation between NPC and infectious keratitis. Moreover, our study considered multiple potential risk factors for the infectious keratitis like the age, sex, systemic diseases, and external eye diseases in the analysis model. The subgroup analyses about infectious keratitis demonstrated that the effect of NPC on infectious keratitis development would not be altered by the presence of radiotherapy or chemotherapy. Consequently, the NPC could be an independent risk factor for the development of infectious keratitis. Additionally, we only included the infectious keratitis episode that occurred one year after the diagnosis of NPC, thus the time sequence between the NPC and subsequent infectious keratitis had been established. The infectious keratitis occurs more frequently in those with thyroid eye disease and exposure keratopathy according to previous literatures [27,28]. Besides, the patients with dry eye disease are prone to develop corneal disorders, such as the corneal erosion and infectious keratitis [29,30]. Thus, the possible explanation for the significant relationship between the NPC and infectious keratitis is that the NPC-induced orbital deformation may contribute to the eyelid disorder and proptosis [19], and leave the cornea to be vulnerable to microbial invasion and infectious keratitis. Although the radiotherapy during the treatment of NPC may cause the corneal epithelial damages which weaken the cornea [31], our study showed that the arrangement of radiotherapy did not elevate the risk of infectious keratitis compared to those without radiotherapy. On the other hand, the cumulative incidence of infectious keratitis in the NPC group revealed a significant trend of increment compared to that in the non-NPC group. This phenomenon could further indicate the effect of NPC with late or advanced stage on the incidence of infectious keratitis. Nevertheless, further study is advocated to confirm this speculation.

In addition to the infectious keratitis, the existence of NPC is correlated to a significantly higher incidence of following orbital cellulitis, but the effect becomes minimal after excluding the influence of radiotherapy. The orbit is a common place of NPC invasion in advanced stage [15,19]. The orbital involvement with severe visual loss could be the first sign of NPC [32]. Besides, a previous study reported a NPC patient with orbital invasion and associated exophthalmos [15]. Accordingly, the invasion of orbit in NPC patients may disrupt the general condition of orbital soft tissue and may let the infection occurs more frequently. However, we found the NPC patients with radiotherapy owned higher aHR of orbital cellulitis compared to NPC patients without radiotherapy. This finding may indicate the prominent effect of radiotherapy on orbital cellulitis development because radiotherapy can damage the ocular structure and may also weaken the orbital region [16]. Still, the overall episodes of orbital cellulitis in the NPC and non-NPC populations were low in which only 35 and 19 cases of orbital cellulitis developed in the NPC and non-NPC groups, which could diminish the statistical power. It is possible that the strict criteria of orbital cellulitis reduced the events in both groups. However, some preseptal cellulitis could be misdiagnosed as orbital cellulitis due to the similar appearance if the computed tomography was not arranged [22,33]. Accordingly, we choose to elevate the accuracy of our diagnosis rather than increase the case numbers, and the incidence of orbital cellulitis is similar to pervious clinical population-based study [34].

Concerning other risk factor for the occurrence of infectious keratitis, the patients aged between 20 to 30 years old were under higher risk of infectious keratitis development compared to the patients aged between 30 to 40 years old. The young population showed a higher incidence of infectious keratitis in previous study [26], and the young population used contact lenses, which is a known risk factor for infectious keratitis, more frequently than the middle-aged population [24,35]. Besides, the allergic respiratory diseases and allergic dermatological diseases correlate to higher probability of infectious keratitis. Maybe the elevated inflammatory statuses in these diseases increase the risk of infectious keratitis [24]. Several external eye diseases, like the dry eye disease and blepharitis, may correlates to the presence of infectious keratitis [36,37]. The two protective factors of infectious keratitis in this study are the male sex and residence in sub-urban area. The male population tends to use less contact lenses than the female population, which may be the reason [26]. The explanation of lower infectious keratitis rate in sub-urban habitat is unclear since people living in sub-urban are not necessarily of poor socioeconomic status [38]. However, the upper limit of 95% CI is close to one, thus the significance may not be clinically important. The insignificant effect of all parameters, except for NPC on orbital cellulitis, could result from the few case numbers.

There are still some limitations in our study. Firstly, the usage of claimed database makes crucial information including TNM stage of NPC, image results of NPC: results of NPC-related laboratory exam, exact dose of radiotherapy in NPC treatment, treatment outcome of NPC, recurrence of NPC, degree of NPC orbital involvement, degree of proptosis or entropion, image results of orbit, microorganism of infectious keratitis and orbital cellulitis, and treatment response of infectious keratitis and orbital cellulitis become unavailable. Additionally, the usage of contact lenses cannot be accessed since most contact lenses in Taiwan are self-made. Besides, the design of the NHIRD/LHID 2000 only allows us to track one outcome at one time. Consequently, whether patients with infectious keratitis developed orbital cellulitis in the following months cannot be accessed. Additionally, we standardized the patient’s general condition, thus the external validity was reduced.

## 5. Conclusions

In conclusion, the existence of NPC is associated with a higher incidence of subsequent infectious keratitis after considering many confounders, including the radiotherapy and chemotherapy. Furthermore, the incidence of infectious keratitis would elevate as the disease interval of NPC increase. On the contrary, the development of orbital cellulitis in NPC was prominently influenced by the arrangement of radiotherapy. Consequently, individuals with advanced or prolonged NPC should be informed about the possible infectious keratitis, and the threshold of referral to ophthalmic department should be decreased. Furthermore, a large-scale prospective study to investigate whether the presence of NPC would diminish the prognosis of infectious keratitis is necessary.

## Figures and Tables

**Table 1:** Characteristics between nasal pharyangeal carcinoma patients and control group.

Characteristics	Non-NPC Group	NPC Group	ASD
N	16,736	4184	
Year of index			0.0000
2001–2005	8010 (47.86%)	2020 (48.28%)	
2006–2010	5168 (30.88%)	1305 (31.19%)	
2011–2015	3558 (21.26%)	859 (20.53%)	
Sex			0.0244
Female	5952 (35.56%)	1537 (36.74%)	
Male	10,784 (64.44%)	2647 (63.26%)	
Age			0.0567
20–30	1349 (8.06%)	326 (7.79%)	
30–40	2914 (17.41%)	687 (16.42%)	
40–50	4485 (26.80%)	1100 (26.29%)	
50–60	4363 (26.07%)	1078 (25.76%)	
60–70	2285 (13.65%)	607 (14.51%)	
>70	1340 (8.01%)	386 (9.23%)	
Urbanization			0.0367
Urban	10,017 (59.85%)	2483 (59.35%)	
Sub-urban	5296 (31.64%)	1335 (31.91%)	
Rural	1423 (8.50%)	366 (8.75%)	
Co-morbidities			
Hypertension	3148 (18.81%)	851 (20.34%)	0.0386
DM	1567 (9.36%)	407 (9.73%)	0.0124
Stable CAD	683 (4.08%)	210 (5.02%)	0.0450
Hyperlipidemia	1529 (9.14%)	424 (10.13%)	0.0338
Cerebrovascular disease	752 (4.49%)	201 (4.80%)	0.0148
Allergic respiratory diseases	1348 (8.05%)	456 (10.90%)	0.0972
Rheumatic disease	132 (0.79%)	46 (1.10%)	0.0321
Allergic otolaryngologic diseases	398 (2.38%)	99 (2.37%)	0.0008
Allergic dermatological diseases	3240 (19.36%)	843 (20.15%)	0.0198
External eye diseases	4070 (24.32%)	1112 (26.58%)	0.0945
Cancer treatment			
Chemotherapy	-	1584 (37.9%)	
Radiotherapy			
Without radiotherapy	-	2328 (55.6%)	
With low dose radiotherapy	-	922 (22.0%)	
With high dose radiotherapy	-	934 (22.3%)	

NPC: nasal pharyngeal carcinoma, N: number, ASD: absolute standardized difference, SD: standard deviation, DM: diabetes mellitus, CAD: coronary arterial disease.

**Table 2:** Outcome between the nasal pharyngeal carcinoma and non-nasal pharyngeal carcinoma participants.

Eye Diseases	Non-NPC Group (N = 16,736)	NPC Group (N = 4184)	*p* Value
Infectious keratitis			
Person months	1,584,236	352,044	
Event	2185	659	
Incidence density ^#^ (95% CI)	1.38 (1.32–1.44)	1.87 (1.73–2.02)	
Crude HR (95% CI)	Reference	1.36 (1.25–1.49) *	
aHR (95% CI)	Reference	1.29 (1.16–1.39) *	<0.0001 *
Orbital cellulitis			
Person months	1,727,918	394,085	
Event	35	19	
Incidence density ^#^ (95% CI)	0.02 (0.01–0.03)	0.05 (0.03–0.08)	
Crude HR (95% CI)	Reference	2.38 (1.36–4.16) *	
aHR (95% CI)	Reference	2.24 (1.17–3.98) *	0.0041 *

NPC: nasal pharyngeal carcinoma, N: number, CI: confidence interval, aHR: adjusted hazard ratio which including demographic and systemic co-morbidities variables. ^#^ Crude incidence rate, per 1000 person months. * Denotes significant difference between the two groups.

**Table 3:** Cox regression for estimate the hazard ratio of infectious keratitis.

Index	aHR (95% CI)	*p* Value
NPC	1.29 (1.16–1.39)	<0.0001 *
Year of index (ref = 2001–2005)		
2006–2010	0.97 (0.88–1.06)	0.7732
2011–2015	0.86 (0.72–1.03)	0.1104
Sex (ref = Female)		
Male	0.74 (0.67–0.78)	<0.0001 *
Age (ref = 30–40)		
20–30	1.32 (1.11–1.52)	0.0006 *
40–50	0.99 (0.86–1.15)	0.8002
50–60	1.02 (0.94–1.16)	0.5398
60–70	0.96 (0.74–1.18)	0.6521
>70	0.76 (0.48–1.17)	0.1944
Urbanization (ref = Urban)		
Sub-urban	0.89 (0.74–0.97)	0.0124 *
Rural	0.85 (0.76–1.02)	0.0505
Co-morbidities		
Hypertension	1.00 (0.90–1.10)	0.9914
DM	1.03 (0.91–1.15)	0.7342
Stable CAD	0.98 (0.81–1.14)	0.8645
Hyperlipidemia	1.01 (0.90–1.16)	0.8477
Cerebrovascular disease	0.85 (0.72–1.06)	0.2022
Allergic respiratory diseases	1.30 (1.14–1.40)	<0.0001 *
Rheumatic disease	1.27 (0.92–1.83)	0.1168
Allergic otolaryngologic diseases	0.98 (0.76–1.21)	0.7826
Allergic dermatological diseases	1.22 (1.11–1.32)	<0.0001 *
External eye diseases	1.48 (1.22–1.97)	<0.0001 *

aHR: adjusted hazard ratio which including demographic and systemic co-morbidities variables, NPC: nasal pharyngeal carcinoma, CI: confidence interval, DM: diabetes mellitus, CAD: coronary arterial disease. * denotes significant correlation between index and infectious keratitis.

**Table 4:** Cox regression for estimate the hazard ratio of orbital cellulitis.

Index	aHR (95% CI)	*p* Value
NPC	2.24 (1.17–3.98) *	0.0041 *
Year of index (ref = 2001–2005)		
2006–2010	0.97 (0.52–1.87)	0.9567
2011–2015	0.30 (0.05–2.01)	0.2341
Sex (ref = Female)		
Male	1.15 (0.69–2.00)	0.6475
Age (ref = 30–40)		
20–30	0.42 (0.09–1.96)	0.3198
40–50	0.92 (0.42–2.09)	0.9142
50–60	0.80 (0.30–2.07)	0.6396
60–70	0.54 (0.17–1.74)	0.2980
>70	0.85 (0.26–3.25)	0.8777
Urbanization (ref = Urban)		
Sub-urban	1.30 (0.74–2.35)	0.4140
Rural	2.10 (0.87–4.92)	0.1735
Co-morbidities		
Hypertension	1.01 (0.46–2.16)	0.9768
DM	1.52 (0.70–3.33)	0.3229
Stable CAD	1.26 (0.40–3.54)	0.6886
Hyperlipidemia	1.32 (0.54–3.01)	0.5681
Cerebrovascular disease	0.89 (0.24–3.10)	0.8826
Allergic respiratory diseases	1.17 (0.56–2.63)	0.7141
Rheumatic disease	1.65 (0.42–6.02)	0.9943
Allergic otolaryngologic diseases	0.79 (0.14–5.53)	0.8642
Allergic dermatological diseases	1.27 (0.69–2.42)	0.4287
External eye disease	1.34 (0.89–3.69)	0.0827

aHR: adjusted hazard ratio which including demographic and systemic co-morbidities variables, NPC: nasal pharyngeal carcinoma, CI: confidence interval, DM: diabetes mellitus, CAD: coronary arterial disease. * denotes significant correlation between index and orbital cellulitis.

**Table 5:** The incidence of infectious eye diseases among patients with nasal pharyngeal carcinoma stratified by chemotherapy and the non-nasal pharyngeal carcinoma population.

Infection Eye Diseases	Non-NPC Group (N = 16,736)	NPC without Chemotherapy (N = 2600)	NPC with Chemotherapy (N = 1584)
Infectious keratitis			
Person months	1,584,236	261,309	90,735
Event	2185	480	179
Incidence density ^#^ (95% CI)	1.38 (1.32–1.44)	1.84 (1.68–2.01)	1.97 (1.70–2.28)
aHR_1_ (95% CI)	Reference	1.33 (1.21–1.47) *	1.43 (1.23–1.66) *
aHR_2_ (95% CI)	0.75 (0.68–0.83) *	Reference	1.07 (0.90–1.28)
Orbital cellulitis			
Person months	1,727,918	292,867	101,218
Event	35	14	5
Incidence density ^#^ (95% CI)	0.02 (0.01–0.03)	0.05 (0.03–0.08)	0.05 (0.02–0.12)
aHR_1_ (95% CI)	Reference	2.36 (1.27–4.39) *	2.44 (0.96–6.22)
aHR_2_ (95% CI)	0.42 (0.23–0.79) *	Reference	1.03 (0.37–2.87)

NPC: nasal pharyngeal carcinoma, N: number, CI: confidence interval, aHR: adjusted hazard ratio which including demographic and co-morbidities variables. ^#^ Crude incidence rate, per 1000 person months. * Denotes significant difference compared with reference group.

**Table 6:** The incidence of infection eye diseases among patients with nasal pharyngeal carcinoma stratified by radiotherapy and individuals without nasal pharyngeal carcinoma.

Infection Eye Diseases	Non-NPC Group (N = 16,736)	NPC without Radiotherapy (N = 2328)	NPC with Low Dose Radiotherapy (N = 922)	NPC with High Dose Radiotherapy (N = 934)
Infectious keratitis				
Person months	1,584,236	229,686	71,126	51,232
Event	2185	442	108	109
Incidence density ^#^ (95% CI)	1.38 (1.32–1.44)	1.92 (1.75–2.11)	1.52 (1.26–1.83)	2.13 (1.76–2.57)
aHR_1_ (95% CI)	Reference	1.40 (1.26–1.55) *	1.10 (0.91–1.34)	1.54 (1.27–1.87) *
aHR_2_ (95% CI)	0.72 (0.65–0.79)	Reference	0.79 (0.64–0.97)	1.11 (0.90–1.36)
Orbital cellulitis				
Person months	1,727,918	259,366	77,800	56,920
Event	35	4	10	5
Incidence density ^#^ (95% CI)	0.02 (0.01–0.03)	0.02 (0.01–0.04)	0.13 (0.07–0.24)	0.09 (0.04–0.21)
aHR_1_ (95% CI)	Reference	0.76 (0.27–2.14)	6.35 (3.14–7.81) *	4.34 (1.70–8.07) *
aHR_2_ (95% CI)	1.31 (0.47–3.70)	Reference	8.33 (2.61–10.57) *	5.67 (1.53–11.21) *

NPC: nasal pharyngeal carcinoma, N: number, CI: confidence interval, aHR: adjusted hazard ratio which including demographic and systemic co-morbidities variables. ^#^ Crude incidence rate, per 1000 person months. * Denotes significant difference compared with reference group.

## Data Availability

The original data is confidential and is preserved by the National Health Insurance Administration. Thus, we cannot provide the data.
